# Fertility desire among HIV-positive women in Tigray region, Ethiopia: implications for the provision of reproductive health and prevention of mother-to-child HIV transmission services

**DOI:** 10.1186/s12905-014-0137-2

**Published:** 2014-11-19

**Authors:** Yohannes Adama Melaku, Ejigu Gebeye Zeleke, John Kinsman, Akberet Kelem Abraha

**Affiliations:** Department of Public Health, Mekelle University, College of Health Sciences, P.O.Box 1871, Mekelle, Ethiopia; Department of Epidemiology and Biostatistics, University of Gondar, Institute of Public Health, Gondar, Ethiopia; Department of Public Health and Clinical Medicine, Umeå Centre for Global Health Research, Umeå University, Umeå, 901 85 Sweden

**Keywords:** HIV-positive women, Fertility desire, Reproductive health, Ethiopia

## Abstract

**Background:**

There is growing recognition of the difficult reproductive decisions faced by HIV-positive women. Studies in both resource-constrained and developed countries have suggested that many HIV-positive women continue to desire children in spite of their understanding of the possible risks that HIV poses. This study investigates the factors associated with fertility desire among HIV-positive women in Tigray region, Ethiopia.

**Methods:**

A cross-sectional survey was conducted among 964 HIV-positive women receiving HIV care in 12 health centers of Tigray region. In each health center, the number of study participants was allocated proportionally to the load of HIV-positive women in the chronic care clinics. A descriptive summary of the data and a logistic regression model were used to identify factors associated with fertility desire using odds ratios with a 95% confidence interval and P-value of 0.05.

**Results:**

Four hundred and thirty nine (45.5%) of the participants reported a desire to have children in the future. Eighty six percent of the women had given birth to at least one live baby at the time of study, with the median number of live births being 2 (Inter quartile range = 1,3). Women in the age group of 15–24 years [AOR = 2.64(95% CI: 1.44, 4.83)] and 25–34 years [AOR = 2.37 (95% CI: 1.60, 2.4 3.50)] had higher fertility desire as compared to women in the age group of 35–49 years. Having no children [AOR = 25.76 (95% CI: 13.66, 48.56)], having one to two children [AOR = 5.14 (95% CI: 3.37, 7.84)] and disclosing HIV status to husband/sexual partner [AOR = 1.74 (95% CI: 1.11, 2.72)] were all independently associated with fertility desire.

**Conclusions:**

Age, HIV disclosure status to husband/sexual partner, and relatively few live children were all found to influence HIV-positive women’s fertility desire. Programmers and policy makers should consider the effects of these factors for HIV-positive women as they develop HIV/AIDS interventions.

## Background

Globally, studies estimate that 75% of all HIV-positive people are of reproductive age. Sub-Saharan Africa is home to60% of all people living with HIV/AIDS, and more than half of these are females [1]. In Ethiopia, for example, the prevalence of HIV/AIDS among women of reproductive age (1.9%) is higher than men (1.0%) in the same age [2]. Thus, interventions to meet the reproductive health needs, and specifically the needs relating to a desire to have children of this population group need to be prioritized as many HIV-positive women continue to want to have babies despite knowledge of their HIV status [3-7].

Evidence indicates that HIV-positive women continue to desire more children in the future, though to differing degrees in different contexts. In two studies conducted in Canada and Malawi, for example, the proportions of HIV-positive women who wanted to have children in the future were 69% and 17%, respectively [4,5]. In Ethiopia, different studies have indicated different levels of fertility desire among HIV-positive women. A study in North Wollo, Ethiopia, demonstrated that 15.7% of HIV-positive women had fertility desire [8]. By contrast, 44% of HIV-positive women in Addis Ababa wanted more children [6], as compared to 53% of women in Harari region [7], and 92% of women in Oromia region, Ethiopia [3]*.* Studies in a number of settings have pointed to different factors which determine the fertility desire of HIV-positive women. For example, young age of HIV-infected individuals is significantly associated with increased fertility desire [4,9-13]. Decreased fertility desire is associated with divorce or separation, as well as having at least one child [4,12-14]. HIV disclosure to a woman’s sexual partner has also been associated with her having increased fertility desire [11,14]. Sero-discordance could also affect fertility desire of couples [15]. A further study indicated that educational status was not significantly associated with fertility desire among HIV-positive women [13], while good perceived health status, and CD4 count more than or equal to 200 cells/mm^3^ were found to be significantly associated with increased fertility desire [14,16].

Evidence about the association between Antiretroviral Therapy (ART) use and fertility desire is mixed, however, with some studies indicating that there is no association between the two [8,13,14,16,17], and others suggesting that commencement of ART was one of the reasons for women’s increased fertility desire, due to an improved quality of life [6,10].

In Ethiopia, a number of studies have been conducted to investigate fertility desire of people living with HIV/AIDS [3,6-8,15,18,19]. However, none of these studies has been conducted on a large scale, incorporating a number of health facilities covering large demographic areas with socio-cultural diversity. The present study addresses this gap in the knowledge base by incorporating a number of health facilities covering all zones of the region, and with a large sample size.

The main aim of this study is to assess the extent of, and the factors associated with fertility desire among HIV-positive women in Tigray region, Ethiopia. This is necessary in order to help stakeholders engaged in Sexual and Reproductive Health programs and policies, such as regional health bureaus and non-governmental organizations working on HIV/AIDS and family planning, to make evidence-based decisions about the interventions they are running. Specifically, it is important to ensure that the necessary resources and programs for improved reproductive health services, such as Prevention of Mother-to-Child Transmission (PMTCT) of HIV and family planning services for HIV-positive women, are in place, and that they are as integrated into AIDS care services as current Regional health policy requires them to be. It is hoped that this will lead to a better quality of life for HIV-positive women, decrease levels of vertical HIV transmission to children, and ultimately reduce maternal and child mortality.

## Methods

### Study setting and design

Tigray region has an estimated total population of just over 4.6 million, of whom 50.7% are female. More than 80% of the population lives in rural areas of the region [20]. In 2012, Tigray had 14 hospitals, 220 health centers and 671 health posts [21].

The 2011 Ethiopian Demographic and Health Survey (EDHS) estimated that overall HIV adult prevalence (15–49 years) in Tigray was 1.8% (2.2% for women and 1.3% for men) [2]. HIV prevalence in Tigray varies considerably across zones, from 0.4% (Central zone) to 2.2% (Western zone) [22].

In 2013, there were 60 health centers in Tigray region which were supported by the Ethiopian Network of HIV/AIDS Treatment, Care and Support (ENHAT-CS). Each of these health centers provided a number of free services, including Voluntary HIV Counseling and Testing (VCT), HIV treatment and care (including ART and PMTCT), and family planning.

This study was conducted at chronic follow-up care clinics (providing both pre-ART support and ART) in 12 ENHAT-CS supported health centers in Tigray region from May to June 2013. The study area is also described elsewhere [23].

The analysis is based on cross-sectional survey data collected from HIV-positive women (including those who are on ART and those who have not yet started treatment) receiving services at ENHAT-CS supported health centers in the region. A medical chart review was also conducted to confirm HIV serology, ART history, and other medical characteristics of the women.

### Eligibility criteria

To be eligible to participate in the study, women were required to be 15–49 years of age, attending a chronic follow-up care ENHAT-CS-supported center, competent to give informed consent, and willing to allow medical record review for the purposes of confirming HIV sero-status and other aspects of their medical history. We considered women to be ART users if they were taking ART at the time of interview. We considered women to be pre-ART (not using ART) if they were not taking ART at the time of survey, or if they were taking ART purely for prophylactic purposes within the context of PMTCT.

### Sample selection

**Figure 1:**
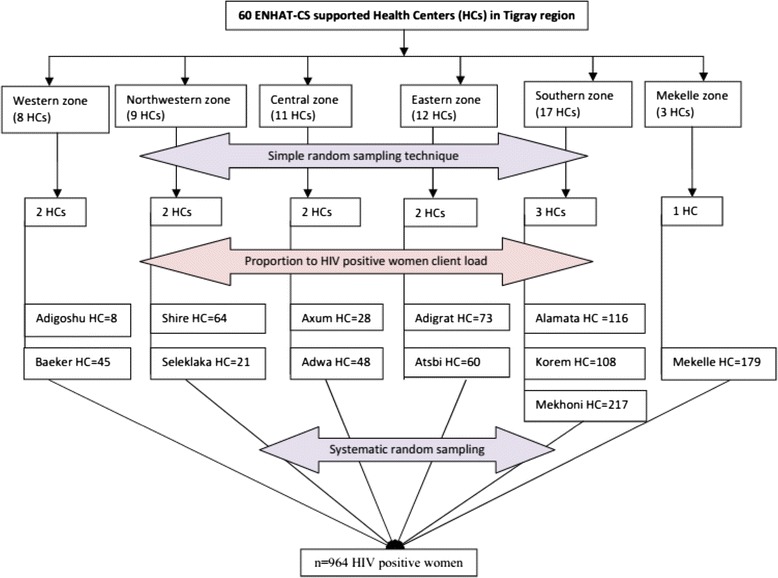
**Schematic presentation of sampling procedure.**

Twenty percent of ENHAT-CS supported health centers in Tigray region were selected with a simple random sampling technique. Around 20% of the health centers in each administrative zone with Tigray (there are 6 zones in the Region) were selected based on the total number of ENHAT-CS supported health centers in each zone. The numbers of study participants in each health center were determined using proportion to population size (HIV-positive women registered in the health centers). To select woman to participate in each health center, a systematic random sampling technique was used. In each health center, every fourth eligible woman was invited to join the study (Figure 1).

### Data collection procedures and tools

After confirming eligibility and obtaining written informed consent, randomly selected participants were asked to complete a 25–35 minute, interviewer-administered questionnaire in the local Tigrenga language.

The questionnaire assessed socio-demographic characteristics, fertility desire, fertility, and sexual history. Questions were adapted from previously conducted studies [4,24-27]. Medical records of HIV-positive women were reviewed to confirm HIV status and other relevant medical history, including date of HIV diagnosis, current WHO stage of disease, most recent CD4 count, ART status, as well as date of ART start, type of ART drugs and other medications/prophylaxis.

### Measures and operational definitions

The explanatory variables included current ART status, age, education, employment, current sexual partnership, number of living children, and HIV-related clinical outcomes. We define *HIV-positive women receiving chronic care* as women who had engaged in care at least once to the selected pre-ART or ART units, whether they were receiving ART or not.

The primary outcome was self-reported fertility desire at the time of the survey. Based on previous similar studies [5,27], the *fertility desire* variable was investigated using the question “Would you like to have children in the future?”, and, the variable was dichotomized into “had no desire” if a woman answered “No”, and “had fertility desire” if she answered “Yes”. If the answer was ‘Yes’, the respondent was asked “How many children would you like to give birth to in the future?”.

### Data quality control, management and analysis

The data were entered into an Epidata version 3.1 database and transferred to STATA version 11.1 (Stata Corporation, College Station, TX, USA) statistical package for analysis.

A pretest of the questionnaire was conducted in two health facilities which had similar services to the selected study health centers. Supervisors checked each completed questionnaire and the data extraction tool (which collected the clinical data listed above) for consistency, completeness and accuracy. Support supervision was also provided by the investigators for data collectors and supervisors.

Frequencies, proportions and summary statistics were used to describe the study population in relation to relevant variables. Person’s *χ*^*2*^, trend *χ*^*2*^ and Fisher’s exact test were used to estimate crude differences among women by different backgrounds in terms of fertility desire. Both bivariate and multivariate logistic regression models were used to identify significant factors associated with fertility desire. The degree of association between independent and dependent variables was assessed using odds ratios with a 95% confidence interval. After testing for co-linearity [28] and interaction [29], all covariates with statistically significant associations in the bivariate analysis were included in multivariate logistic regression model to obtain adjusted estimates of the association between covariates and fertility desire. All statistical tests were two-sided and considered statistically significant at P-value of 0.05.

### Ethics statement

Ethical clearance was obtained from the Institutional Review Board of Mekelle University, College of Health Sciences. Permission letters were gained from Tigray regional Health Bureau, respective district health offices, and the in-charges of the participating health centers. After explaining the objective and contents of the study, written consent was obtained from each respondent, or from the adult next-of-kin when the respondent was under 18 years of age. The information collected was purely used for research works and all respondents have remained anonymous.

## Results

### Socio demographic, clinical and fertility characteristics of the study participants

All 964 eligible study participants gave responses during the interview, and allowed their medical records to be reviewed – this gave a response rate of 100%. The median age was 30 years (Inter Quartile Range (IQR) = 27, 36). More than two-thirds (68.8%) of the participants were from urban areas. A large majority, 92.6% and 91.8%, of the participants were ethnic “Tigrie” and orthodox Christians, respectively. Three-fifths (59.1%) of the women were illiterate. Housewives, daily laborers and farmers collectively accounted for more than three-fifths (20.1%, 21.7% and 20.2%, respectively) of all the occupations of the study participants. Exactly two-fifths (40%) of the participants were married at the time of survey. More than 69% and 57%, respectively, of the participants had no functional television and radio in their house (Table 1).

**Table 1:** **Socio demographic, clinical and fertility characteristics of HIV-positive women of reproductive age in Tigray region, Ethiopia, 2013 (N = 964 unless specified)**

		**Had fertility desire**		
**Characteristics**	**Frequency (%)**	**Yes (%)**	**No (%)**	**P-value**
**Age category in years**				<0.0001**
15-24	120(12.5)	84(70.0)	36(30.0)	
25-34	508(52.7)	268(52.8)	240(47.2)	
35-49	336(34.9)	87(5.9)	249(74.1)	
**Median (IQR)**	30(27, 36)			
**Residence**				0.160*
Urban	663(68.8)	312(47.1)	351(52.9)	
Rural	301(31.2)	127(42.2)	174(57.8)	
**Ethnicity**				0.680*
Tigrie	893(92.6)	405(45.4)	488(54.7)	
Other (Amhara, Oromo and Afar)	71(7.4)	34(47.9)	37(52.1)	
**Religion**				0.343*
Orthodox	885(91.8)	399(45.1)	486(54.9)	
Other (Muslim, Catholic and Protestant)	79(8.2)	40(50.6)	39(49.4)	
**Maternal education**				
No formal education	570(59.1)	225(39.5)	345(60.5)	0.0001**
Primary education	257(26.7)	141(54.9)	116(45.1)	
Secondary education and above	137(14.2)	73(53.3)	64(46.7)	
**Occupation**				<0.0001*
Unemployed	117(12.1)	41(35.0)	76(65.0)	
Housewife	194(20.1)	100(51.6)	94(48.5)	
Daily laborer	209(21.7)	91(43.5)	118(56.6)	
Farmer	195(20.2)	72(36.9)	123(63.1)	
Merchant	134(13.9)	72(53.7)	62(46.3)	
Employed	75(7.8)	34(45.3)	41(54.7)	
Other	40(4.2)	29(72.5)	11(27.5)	
**Marital status**				<0.0001*
Never Married	66(6.9)	44(66.7)	22(33.3)	
Married/partnered	463(48.0)	251(54.2)	212(45.8)	
Dissolved (widowed/divorced)	435(45.1)	144(33.1)	291(66.9)	
**Presence of functional television in the house**				0.001*
Yes	297(30.8)	160(53.9)	137(46.1)	
No	667(69.2)	279(41.8)	388(58.2)	
**Presence of functional radio in the house**				0.004*
Yes	415(43.1)	211(50.8)	204(49.2)	
No	549(57.0)	228(41.5)	321(58.5)	
**HIV status disclosed to anyone**				0.078*
Yes	822(85.3)	384(46.7)	438(53.3)	
No	142(14.7)	55(38.7)	87(61.3)	
**Did you disclose to your husband/sexual partner? (n = 822)**				<0.0001*
Yes	418(50.9)	221(52.9)	197(47.1)	
No	546(49.1)	163(40.4)	241(59.6)	
**Perceived current health status**				0.112^#^
Deteriorating	15(1.5)	3(20.0)	12(80.0)	
Improving	897(93.1)	410(45.7)	487(54.3)	
Same	52(5.4)	26(50.0)	26(50.0)	
**Current WHO clinical stage**				0.5378**
I	558(57.9)	255(45.7)	303(54.3)	
II	171(17.7)	84(49.1)	87(50.9)	
III	214(22.2)	91(42.5)	123(57.5)	
IV	21(2.2)	9(42.9)	12(57.1)	
**Last CD4 count (n = 930) in cells/mm** ^**3**^ **(n = 930)**				0.4926**
<200	163(17.5)	70(42.9)	93(57.1)	
> = 200	767(82.5)	352(45.9)	415(54.1)	
Median CD4 count (IQR)	351 (IQR = 235, 521)			
**Currently on ART**				0.543*
Yes	822(85.3)	371(45.1)	451(54.9)	
No	142(14.7)	68(47.9)	74(52.1)	
**Taking other medication/prophylaxis currently**				0.011*
Yes	760(78.8)	330(43.4)	430(56.6)	
No	204(21.2)	109(53.4)	95(46.6)	
**Know husband’s/partner’s HIV status (n = 463)**				
Yes	371(80.1)	205(55.3)	166(44.7)	0.365*
No	92(19.9)	46(50.0)	46(50.0)	
**Husband’s/partner’s HIV status (n = 371)**				0.451*
Negative	85(22.9)	50(58.8)	35(41.2)	
Positive	286(77.1)	155(54.2)	131(45.8)	
**Total number of living children**				0.0001**
None	167(17.3)	132(79.0)	35(21.0)	
1-2	508(52.7)	260(51.2)	248(48.8)	
> = 3	289(30.0)	47(16.3)	242(83.7)	
Median number of living children (IQR)	2(IQR = 1,3)			

*-Person χ^2^ test, **-Trend χ^2^ test, ^#^-Fisher’s exact test.

The level of HIV disclosure among the women was high, with 822 (85.3%) reporting they had informed at least one person of their HIV-positive status. However, almost half of these women, 414 (49.1%), had not disclosed to their husband/sexual partner. A large majority, 897 (93.1%), of the women reported that their health status was improving. Of these, 789 (88%) were on ART. In all, 822 (85.3%) of the women were on ART at the time of survey (Table 1).

More than half of the women, 558 (57.9%), were in WHO clinical stage I, and more than four-fifths of them, 767 (82.5%), had greater than or equal to 200 cells/mm^3^ CD4 count in their latest laboratory result. Of those who were married or partnered at the time of survey, 92(19.9%) did not know their husband’s/partner’s HIV status. Nearly four-fifths, 286 (77%), of the women who knew their partner’s/husband’s HIV status, reported that he is HIV-positive. The majority, 508 (52.7%), of women had one or two living children with the median number of children being 2 (IQR = 1, 3) (Table 1).

Fertility desire was crudely associated with age and women’s education status (P < 0.0001). There was also association between marital status and women’s fertility desire (P < 0.0001). Associations were detected between fertility desire of women and the presence of television and radio. Disclosure of HIV status and total number of living children a mother had were found to be significantly associated with fertility desire (P < 0.0001) (Table 1).

Thirty nine (3.5%) of the women reported that they were pregnant, and of these, five (14.7%) said that their pregnancy was unplanned and unwanted. Four hundred thirty nine (45.5%) of all the HIV-positive women expressed a desire to have more children in the future. Of the women who did not desire to have children and who were sexually active at the time of the survey (n = 453), 182 (40.2%) were not using any contraceptive methods.

Half (49.7%) of the women who did not report any desire to have children (n = 525; 54.5%) reported that this was because they did not have enough income to support more children. The other reasons given were the presence of enough children (44.2%), fear of further deterioration of health (24.2%), fear of transmission of HIV to the child (14.1%), and advice from health professionals (6.7%). Partner objection (0.8%) and other reasons (5.3%) were also mentioned.

### Factors associated with fertility desire

Of all the variables modeled in a bivariate logistic regression, marital status, age, education level, occupation, presence of functional television and radio, total number of living children, HIV status disclosure to husband/sexual partner, perceived health status, and being on Cotrimoxazole prophylaxis or non-ART medication were found to be significantly associated with fertility desire at P-value of 0.05% (Table 2).

**Table 2:** **Factors associated with fertility desire among HIV-positive women of reproductive age in Tigray region, Ethiopia, 2013**

		**Had fertility desire**				
**Characteristics**		**Yes (%)**	**No (%)**	**COR (95% CI)**	**AOR (95% CI)**	**P-value**
**Marital status**	Married/partnered	251(54.2)	212(45.8)	0.59(0.34, 1.02)	0.94(0.40, 2.21)	0.889
	Dissolved (widowed or divorced)	144(33.1)	291(66.9)	0.25(0.14, 0.43)	0.61(0.29, 1.28)	0.189
	Never married	44(66.7)	22(33.3)	1.00	1.00	
**Age category in years**	15-24	84(70.0)	36(30.0)	6.68(4.21, 10.58)	2.64(1.44, 4.83)	0.002
	25-34	268(52.8)	240(47.2)	3.20(2.37, 4.31)	2.37(1.60, 3.50)	<0.0001
	35-49	87(5.9)	249(74.1)	1.00	1.00	
**Educational status**	No formal education	225(39.5)	345(60.5)	1.00	1.00	
	Primary education	141(54.9)	116(45.1)	1.86(1.38,2.51)	1.16(0.77, 1.76)	0.476
	Secondary education and above	73(53.3)	64(46.7)	1.75(1.20,2.55)	0.81(0.45, 1.45)	0.474
**Occupation**	Unemployed	41(35.0)	76(65.0)	1.00	1.00	
	Housewife	100(51.6)	94(48.5)	1.97(1.23,3.16)	1.58(0.75, 3.31)	0.226
	Daily laborer	91(43.5)	118(56.6)	1.43(0.90,2.28)	1.47(0.77, 2.84)	0.246
	Farmer	72(36.9)	123(63.1)	1.09(0.67,1.75)	1.85(0.95,3.61)	0.072
	Merchant	72(53.7)	62(46.3)	2.15(1.29,3.58)	2.38(1.17, 4.84)	0.017
	Employed	34(45.3)	41(54.7)	1.54(0.85,2.78)	1.11(0.48,2.60)	0.807
	other	29(72.5)	11(27.5)	4.89(2.22,10.78)	5.38(1.64,17.62)	0.005
**Presence of functional television**	Yes	160(53.9)	137(46.1)	1.62(1.23,2.14)	1.07(0.70,1.63)	0.768
	No	279(41.8)	388(58.2)	1.00	1.00	
**Presence of functional radio**	Yes	211(50.8)	204(49.2)	1.46(1.13,1.88)	1.39(0.96,2.03)	0.082
	No	228(41.5)	321(58.5)	1.00	1.00	
**Did you disclose to your husband/sexual partner?**	Yes	221(52.9)	197(47.1)	1.66(1.26, 2.19)	1.74(1.11,2.72)	0.016
	No	163(40.4)	241(59.6)	1.00	1.00	
**Perceived health status**	Deteriorating	3(20.0)	12(80.0)	1.00	1.00	
	Improving	410(45.7)	487(54.3)	3.37(0.94,12.01)	3.91(0.72,21.29)	0.115
	The same	26(50.0)	26(50.0)	4.00(1.01,15.85)	4.37(0.67,28.41)	0.122
**On prophylaxis/non-ART medication**	Yes	330(43.4)	430(56.6)	1.00	1.00	
	No	109(53.4)	95(46.6)	1.50(1.10,2.04)	1.26(0.82,1.92)	0.289
**Total number of living children**	None	132(79.0)	35(21.0)	19.41(11.94, 31.58)	25.76(13.66, 48.56)	<0.0001
	1-2	260(51.2)	248(48.8)	5.40(3.78, 7.72)	5.14(3.37,7.84)	<0.0001
	> = 3	47(16.3)	242(83.7)	1.00	1.00	

COR-Crude Odds Ratio, AOR-Adjusted Odds ratio, CI-Confidence Interval.

In a multivariate analysis, women in the age groups of 15–24 and 25–34 years had 2.6 [AOR = 2.64 (95% CI: 1.44, 4.83)] and 2.4 [AOR = 2.37 (95% CI: 1.60, 3.50)] higher odds of fertility desire compared to women in the age group of 35–49 years, respectively. Women who has disclosed their HIV status had almost two times [AOR = 1.74 (95% CI: 1.11, 2.72)] higher odds of fertility desire compared to women who had not. Women who had no children had 26 times [AOR = 25.76 (95% CI: 13.66, 48.56)] higher odds of desiring children as compared to women who already had three or more children; and women who already had one or two children had five times [AOR = 5.14 (95% CI: 3.37, 7.84)] higher odds of desiring more children (Table 2).

## Discussion

In this study, 45% of the HIV-positive women we surveyed desired to have children in the future. This finding is lower than findings from similar studies in Swaziland, 60% [24]; Uganda, 59% [9] and 72% [30]; and Canada, 69% [4]; while it is higher than the figures derived from studies conducted in Tanzania, 36% [14]; Uganda, 10% [12], 19% [31], and 29% [11] and Malawi, 17% [5]. In Ethiopia, various studies have reported different level of fertility desire. A similar level was reported in Addis Ababa (44%) [6]; a lower level was reported in Northern Ethiopia (24%) [18] and South Wollo (15.7%) [8]. Higher rate of fertility intention (92.3%) was also reported in Oromia region [3]*.* A study in Harari region indicated that 52.9% of women had fertility desire [7]. The reasons for these differences are not clear, but they are likely to be the products of specific socio-cultural and/or economic factors – or the differential expressions of these – in each setting.

Our findings indicated that ART use was not significantly associated with fertility desire, and neither was CD4 count, even though both of these would have led to or indicated improved health. These findings were consistent with studies (including a meta-analysis) conducted in Tanzania, Ethiopia, Malawi [8,13,14,16,17]. The important issue, therefore, may not have been the women’s clinical status, but rather, perhaps, their socio-demographic characteristics and fertility history.

Other studies have found the factors predicting fertility desire to include, for example, the desire to have children of both sexes, the desire for a large family, pressure from family or partner to have children [32], and a child being thought of as a prerequisite for a fulfilled and happy life [33]. The most important factors predicting fertility desire in our study population, by contrast, included age, disclosing HIV status to husband/sexual partner, and having no or few living children.

After adjusting for other variables, we found that age of women was significantly associated with fertility desire. Women of younger age had higher odds fertility desire compared to older women. This finding is similar from those of a methodologically diverse series of studies from Uganda and Canada, which indicate that young, HIV-infected women experience significantly more fertility desire than HIV-infected, older women [4,9-12]. In Ethiopia, some studies demonstrated the presence of an association between age and fertility desire [6,7], while another study reported that such an association was not present [8].

In the current study, women who disclosed their HIV status to their husband/sexual partner had higher odds of having fertility desire. HIV disclosure to a woman’s sexual partner has also been associated with her having increased fertility desire [11,14]. A study in Ethiopia has reported the same finding [8], probably because communication between partners may play an important role in pre-conception planning behaviors [34]. Additionally, it is important to understand the positive impact of communication on partner participation in PMTCT programmes [35,36]. Thus, interventions to promote the reproductive health of HIV-positive women in Ethiopia should continue to promote positive couple communication about HIV status.

Our finding that women who had no children, or who had one or two children, were much more likely to desire babies than women who had more than two children, was also expected: similar findings have been reported in a number of different countries [4,12-14,17,37], including in Ethiopia [6,7,16,19]. This could be that in some communities, such as Ethiopia, a child is thought of as a prerequisite for a fulfilled and happy life [33].In some African communities, satisfying desired family size, desire for biological children, maintaining stability of the union, and socio-cultural pressures were important reasons for continuing fertility desire among HIV-positive women [38].

An important finding of our study was that slightly more than half of these HIV-positive women reported that they did not want to have children in the future. Several reasons were given for this, with two of the most important being insufficient financial security and a fear of further deterioration in their health [10]. In other words, these women may have wanted children had their financial and health circumstances been better, a finding which may also be reflected in the wider HIV-negative female population in the region. Counseling on safer contraceptive methods for these women will have important reproductive health implications.

The current study found that 40.2% of HIV-positive women had an unmet need for contraceptives. This represents a potentially serious public health problem. It is essential that the contraceptive needs of these women are met, so that they may avoid the anxieties and possible health risks associated with an unwanted pregnancy. The health workers providing their chronic AIDS care must be made aware of this, and a range of contraceptive options should be made available, as well as all the associated information about these options.

## Study limitations

It is important to note some limitations of this study. First, men were not included in the study, in spite of the essential role they can play in deciding about the size of their family. Second, health service providers, whose attitudes and perceptions could have a direct or indirect influence on fertility desire in this population, were not also included. Structural components of the health system and service provision quality would also be important considerations in future studies on this topic.

This study is also limited to women who are receiving chronic, long term care for their HIV infection. Fertility desire among these women could be very different from women who are not receiving such care. In addition, small numbers of HIV-positive women who are not ART users were not specifically powered to detect differences in terms of their fertility desire. Lack of comparison to HIV-negative women may also be seen as a limitation.

## Conclusions

This study has identified two discrete groups of HIV-positive women in Tigray region, Ethiopia, with very clear and differing requirements from the health services. One of these groups, constituting 45% of the 964 women surveyed, report that they want to have children in the future; while the other group, made up of the remaining 55% of the women, say that they do not want to have children. The factors associated with fertility desire in the former group included being young, having no children, and disclosing HIV status to their husband/sexual partner; while the primary reasons for the women in the latter group not wanting to have children were insufficient financial security, having ‘enough’ children already, and a fear of deteriorating health.

These findings have important implications for sexual and reproductive health policy and practice in Tigray. Tigray regional SRH policy stipulates that services should be fully integrated into existing HIV/ART care and it is important that this integration is maintained and strengthened in practice. This strengthening could include ensuring that health workers are well informed about the possible effect of the factors identified in this study that are associated with HIV-positive women’s desire either to have children or not. Full information about the possible risks of having a baby should be provided to all HIV-positive women, but in a way that protects their reproductive rights, and ensures that any decision to conceive is supported all the way through the pregnancy, birth, and post-natal period. Meanwhile, for those HIV-positive women who do not desire to have children, a range of family planning method options should be made accessible from all HIV/AIDS care units.

Further research on this topic should include qualitative studies that would provide a deeper understanding of HIV-positive women’s fertility desires. One important aspect of this would be the possible role of health care workers in ensuring that the rights of HIV-positive women are protected, and that their desires and decisions to have children, or not to have children, are supported. In addition, further studies should consider including a comparison with HIV-negative women.
